# Biodegradation of Cholesterol by *Cellulosimicrobium cellulans* YS01 Isolated from the Gut of Healthy Individuals

**DOI:** 10.3390/microorganisms13071451

**Published:** 2025-06-22

**Authors:** Panqi Sheng, Qianqian Xu, Kaige Zhang, Xiaoyu Cao, Xinyue Du, Kun Lin, Hai Yan

**Affiliations:** School of Chemistry and Biological Engineering, University of Science and Technology Beijing, Beijing 100083, China; m202321061@xs.ustb.edu.cn (P.S.);

**Keywords:** cholesterol, *Cellulosimicrobium cellulans* YS01, genome analysis, biodegradation

## Abstract

An excessively high serum cholesterol (CHOL) level in humans can easily lead to cardiovascular diseases (CVDs), including hypertension and coronary heart disease. In this study, a CHOL-lowering bacterium, *Cellulosimicrobium cellulans* YS01, was isolated from healthy human intestinal microbiota and identified via average nucleotide identity (ANI) analysis. The cells of YS01 degraded 74.00% of CHOL within 5 d, which decreased from the initial 1.00 g/L to 0.26 g/L. And its extracellular crude enzymes achieved equivalent efficiency within 24 h, which decreased from the initial 0.50 g/L to 0.13 g/L. The results indicated that YS01 indeed has a strong ability in the biodegradation of CHOL. Furthermore, the whole genome analysis of YS01 revealed that cholesterol oxidase and choloylglycine hydrolase encoded by gene *choD* and gene *cbh*, respectively, may play key roles in the conversion of CHOL. Cholest-4-ene-3-one was produced from CHOL through the catalysis by cholesterol oxidase, and choloylglycine hydrolase was also involved in another biodegradation pathway of CHOL. The results provide scientific insights into the mechanisms of biodegrading CHOL using *C. cellulans* YS01 and lay a solid foundation for the development of new CHOL-lowering strategies based on microbial therapy.

## 1. Introduction

Cholesterol (CHOL) serves critical physiological roles, functioning as a structural component of cellular membranes [1], a precursor for steroid hormone biosynthesis, and a mediator in vitamin D3 activation and bile acid synthesis pathways [2]. Cardiovascular diseases (CVDs), particularly hypercholesterolemia and atherosclerosis, demonstrate strong pathophysiological correlations with systemic CHOL homeostasis. Currently accounting for 46.20% of total mortality in China [3], cardiovascular diseases (CVDs) are predominantly driven by dyslipidemia-induced vascular pathogenesis [4]. Hypercholesterolemia is an independent CVD risk factor, contributing to atherosclerosis and ischemic events through the accumulation of low-density lipoprotein (LDL) CHOL in arterial walls [5].

Current pharmacological interventions, such as statins targeting 3-hydroxy-3-methylglutaryl coenzyme A (HMG-CoA) reductase, effectively reduce CHOL synthesis but are associated with adverse effects including myopathy and hepatotoxicity [6], which have intensified efforts to explore safer alternatives, particularly those leveraging the gut microbiota’s metabolic potential to modulate CHOL homeostasis [7].

Cholesterol oxidase, also known as 3β-hydroxycholesterol oxidase, serves as a catalyst in the initial step of CHOL or other natural sterol biodegradation. As a dual-functional flavin monooxygenase, it catalyzes the oxidation of steroid substrates, with its enzymatic activity strictly dependent on the essential presence of a hydroxy group at the 3β-position of the steroid ring structure [8]. The microorganisms currently reported to produce cholesterol oxidase primarily include the species *Nocardia*, *Pseudomonas*, *Brevibacterium*, *Rhodococcus*, *Streptomyces*, *Arthrobacterium*, *Corynebacterium*, and *Bacillus* [9]. Cholesterol oxidase can catalyze the oxidation of CHOL into cholest-4-en-3-one, serving as a valuable analytical tool for detecting CHOL levels, which is widely utilized in the assessment of atherosclerosis, thrombosis, CVDs, and other lipid-related disorders [10].

The gut microbiome is a dynamic ecosystem that exerts profound effects on host lipid metabolism through enzymatic transformations, metabolite signaling pathways, and competitive inhibition of CHOL absorption [11]. Some bacterial strains such as *Enterococcus faecium* GEFA01 have been demonstrated to exhibit CHOL-lowering effects via promoting reverse CHOL transport and regulating the generation of short-chain fatty acids (SCFA) [12]. Currently, research on biodegradation of CHOL has primarily focused on lactic acid bacteria (LAB), which are predominantly probiotic bacteria [13]. Most LAB possess bile salt hydrola (BSH) activity and exhibit acid tolerance and bile-salt tolerance, enabling them to overcome the harsh environments of the stomach and intestines, thereby facilitating colonization in the intestinal tract [14]. The activity of BSH is considered a critical factor in reducing CHOL levels and thus serves as a primary selection marker for LAB with CHOL-lowering properties [15,16]. While LAB exhibits strong CHOL removal capabilities, it is highly sensitive to environmental changes and demonstrates poor stress resistance [17].

Muhammed Majeed et al. [18] investigated *Bacillus coagulans* MTCC 5856, a strain capable of biodegrading CHOL in vitro. Under simulated physiological conditions, this probiotic significantly reduced CHOL levels in CHOL-rich foods, including egg yolks (39.79%), chicken livers (45.44%), and butter (49.51%). Furthermore, SCFA were detected in metabolic products, which may inhibit CHOL biosynthesis in the liver [19]. These findings provide conclusive evidence for the nutritional therapeutic potential of *B. coagulans* in managing dietary-induced hypercholesterolemia. Kokila et al. [20] constructed in vitro 3T3-L1 adipocytes and high-fat diet-induced rat models to investigate the CHOL-lowering capability of *Bacillus amyloliquefaciens* KAVK1, which achieves CHOL clearance by biodegrading the side chains of CHOL molecules, and simultaneously produces lipase inhibitors to reduce fat digestion and absorption. Therefore, there is a pressing need to develop novel bacterial strains with highly efficient CHOL-biodegrading capabilities.

In this study, we successfully isolated a *C. cellulans* YS01 strain from healthy human intestinal microbiota, which exhibits a significant ability to biodegrade CHOL. Both the cells and extracellular enzymes of YS01 have demonstrated a strong ability to degrade CHOL. The genomic analysis of YS01 provided information such as genomic characteristics and functional annotations, revealing that cholesterol oxidase and choloylglycine hydrolase encoded by gene *choD* and gene *cbh*, respectively, may play a key role in the process of CHOL biodegradation. Essentially, our study aims not only to reveal the CHOL-degrading mechanism of YS01, but also to provide a reliable basis for the further development of strategies based on microbial therapy. Future research should focus on in vivo experiments of YS01, and it is crucial to study its specific mechanism in vivo.

## 2. Materials and Methods

### 2.1. Materials and Culture Media

Cholesterol (≥99% purity) was commercially sourced from Macklin Biochemical Technology Co., Ltd. (Shanghai, China). All other reagents used for this analysis are chromatographic grade. *Cellulosimicrobium cellulans* YS01 was isolated from the gut of healthy individuals.

The basic liquid screening medium is composed of the following components: 0.5 g/L KH_2_PO_4_, 0.5 g/L NH_4_Cl, 0.5 g/L Na_2_CO_3_, 0.1 g/L MgSO_4_, and 1.0 g/L CHOL. Among them, cholesterol is utilized as the sole carbon source. LB liqid medium was used to culture YS01, which was composed of the following: 10.0 g/L tryptone, 5.0 g/L yeast powder, and 10.0 g/L NaCl. Solid media were prepared by adding 18–20 g/L agar to the liquid medium. The above-mentioned culture media were all sterilized at 121 °C for 20 min and then used in the subsequent experiments.

### 2.2. Isolation of Bacterial Strain for Biodegrading CHOL

The processed human gut microbiota samples were inoculated into 20 mL of the basic liquid screening medium and incubated at 37 °C for 5 d under continuous shaking. Subsequently, under the same culture conditions, 1.0 mL of the culture was transferred into the fresh liquid screening medium, every 5 d being regarded as a cycle. After 25 d, the last culture was dilution-plated on LB agar and incubated at 37 °C for 3 d to obtain monoclonal colonies. Isolated colonies from LB plates were inoculated into basal liquid medium to assess biodegradative capability. All stages adhered to strict aseptic protocols.

### 2.3. Genome Sequencing of YS01

DNA was extracted using MagPure Bacterial DNA Kit (D6361-02, Magen, Guangzhou, China), with concentration quantified via Qubit4.0 (Thermo (Waltham, MA, USA), Q33226). DNA integrity was verified by 1% agarose gel electrophoresis. After random fragmentation of genomic DNA (with an average size of 200–400 bp), fragments were selected for end repair, 3′-adenylation, adapter ligation, and polymerase chain reaction (PCR) amplification. Post-purification with magnetic beads, libraries underwent dual qualification: fluorometric quantification (Qubit 4.0) and length distribution analysis (2% agarose gel electrophoresis). Finally, the eligible libraries were sequenced on the Illumina NovaSeq 6000 platform at Sangon Biotech (Shanghai, China).

Raw sequencing reads were processed with Trimmomatic v0.36 [21] to remove adapter contamination and trim low-quality segments, then high-fidelity reads were obtained. Genome assembly was accomplished using SPAdes v3.15 [22], and the Gapfiller v1.11 [23] was used to fill gaps. Gene predictions and annotations were performed using the Prokka Version 1.10 [24] and the National Center for Biotechnology Information (NCBI) database. Based on protein-coding genes, the gene functions were annotated using the Cluster of Orthologous Groups of proteins (COG) [25] and the Kyoto Encyclopedia of Genes and Genomes (KEGG) [26] database.

### 2.4. Analysis of CHOL and Its In Vitro Biodegradation with HPLC

The CHOL was analyzed by high performance liquid chromatography (HPLC, Shimadzu LC-20AT, Tokyo, Japan). The culture was centrifuged at 12,000 rpm for 20 min, then the supernatant was discarded. Next, 20 mL of methanol was added to the centrifuge tube, and the vortex mixing treatment was used for 10 min to ensure full dissolution. After filtering the sample through an organic phase microporous membrane (0.22 μm), 40 μL was aspirated and detected by HPLC. The chromatographic column was Agilent ZORBAX SB-Aq (Agilent, Santa Clara, CA, USA) (150 mm × 4.6 mm, 5 µm). The mobile phases included solvent A 50% methanol and solvent B 50% methanol. The elution method was binary high-pressure elution. Cholesterol was detected at an ultraviolet detection wavelength of 206 nm, the flow rate was 1 mL/min, and the column oven temperature was controlled at 35 °C [27]. The standard curve for CHOL was determined by HPLC, and the correlation coefficient (R^2^) was 0.9994, indicating a good correlation. The peak area was linearly fitted against the concentration of CHOL in standard working solutions. Based on the established calibration curve, the content of CHOL in bacterial solution samples was calculated, and the biodegradation rate of CHOL was obtained.

### 2.5. Biodegradation of CHOL by YS01

The YS01 was inoculated into 20 mL of LB liquid medium and incubated at 37 °C with shaking for 48 h. Subsequently, the culture of YS01 was transferred to the basic screening medium at an inoculation volume of 1% and incubated at 37 °C with the shake rate of 200 rpm for 5 d. The basic screening medium without YS01 inoculation was used as the negative control and cultured under the same conditions. Samples from different periods were preserved for subsequent testing. The content of CHOL was measured by HPLC and the degradation rate was calculated.

### 2.6. Biodegradation of CHOL by Extracellular Crude Enzymes of YS01

The YS01 was inoculated into 20 mL of LB liquid medium and incubated at 37 °C with shaking for 48 h. The extracellular enzymes were collected from YS01 culture medium via centrifugation at 12,000 rpm for 20 min and subjected to a measurement of the optical density at 562 nm [28]. The content of extracellular crude enzyme protein was determined using ultraviolet (UV) spectrophotometry, and the enzyme activity was characterized according to the definitions of enzyme activity and specific activity [29]. Next, the extracellular crude enzymes were transferred into centrifuge tubes with an initial CHOL concentration of 0.50 g/L and further cultured at 37 °C with a shake rate of 200 rpm for 24 h. Centrifuge tubes without the extracellular enzymes were used as the negative control. During the culture process, samples from different periods were preserved for the determination using HPLC.

### 2.7. Statistical Analysis

All experiments were conducted in triplicate, and the results are expressed as mean ± standard deviation (Means ± SD). Statistical analyses were performed using OriginPro 2025 (Originlab, https://www.originlab.com (accessed on 10 May 2025)). Analysis of variance (ANOVA) was performed using IBM SPSS statistical (version 27.0, SPSS Inc., Chicago, IL, USA) software to validate significance.

## 3. Results

### 3.1. Isolation and Identification of YS01

**Figure 1:**
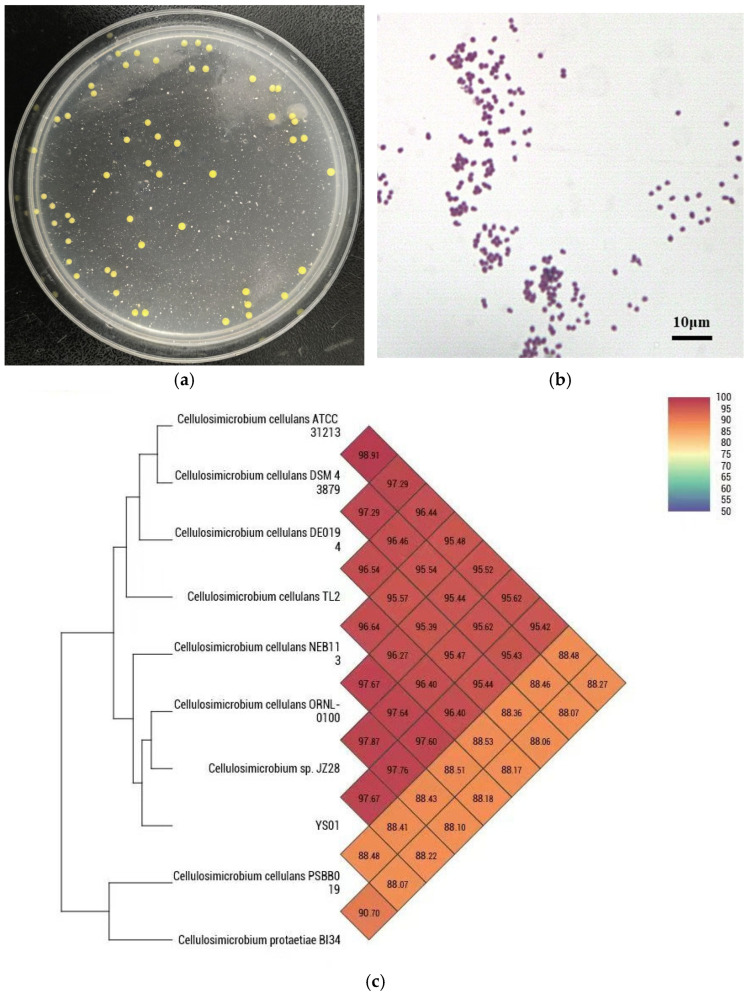
(**a**) Colonies morphology of YS01. (**b**) Morphology of YS01 under the light microscope. (**c**) Heatmap of average nucleotide identity.

The monoclonal colonies of YS01 were formed on agar plates (Figure 1a), with a golden yellow colour, circular shape, smooth and moist surface, clear edges, and diameters ranging from 1 to 2 mm. The cells of YS01 were observed to be Gram-positive and spherical under the light microscope (Figure 1b). Analysis through 16S rRNA gene sequencing confirmed that YS01 belongs to the genus *Cellulosimicrobium*. According to average nucleotide identity (ANI) testing, the genomic homology between YS01 and the genome of *Cellulosimicrobium cellulans* is 97.76% (Figure 1c). The recommended cutoff values for species delineation by OrthoANI and raw ANI are 95~96%. Therefore, it can be concluded that strain YS01 belongs to the species *Cellulosimicrobium cellulans*.

### 3.2. Biodegradation of CHOL by YS01 and Extracellular Crude Enzymes

**Figure 2:**
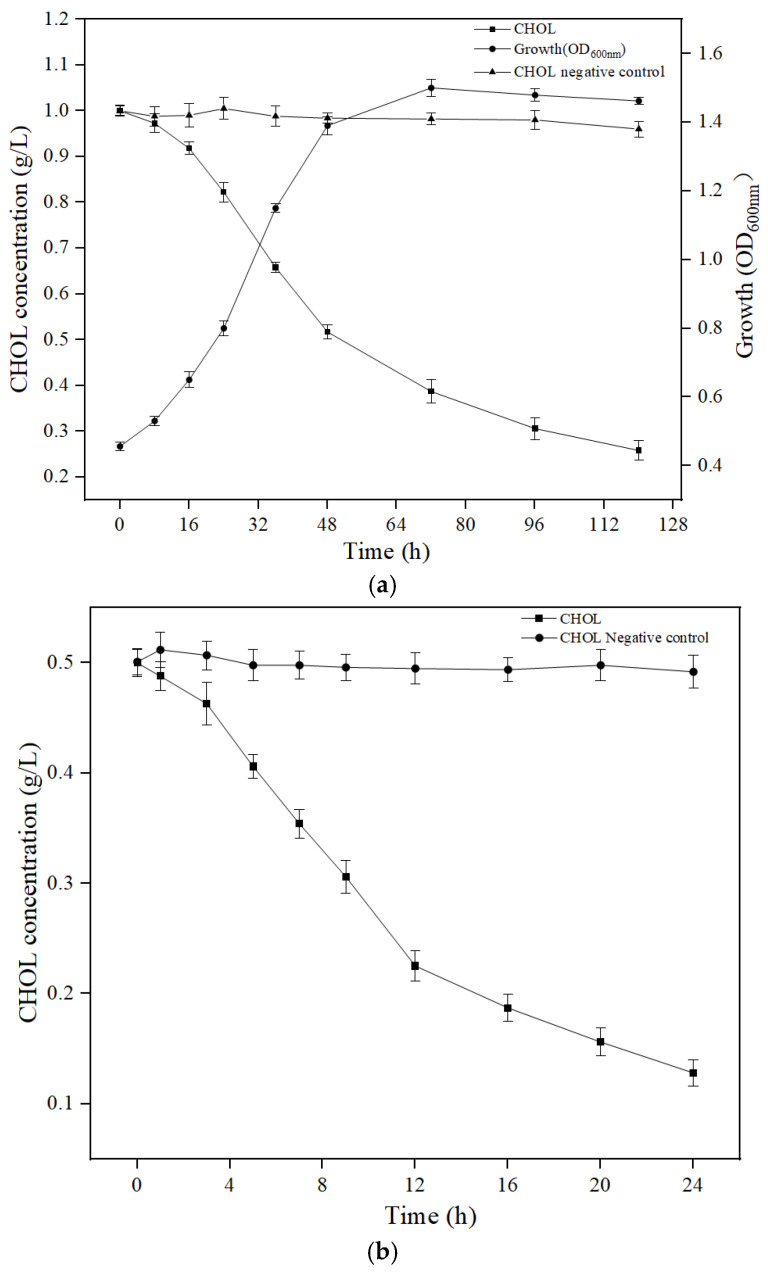
(**a**) Growth and biodegradation kinetics of CHOL of YS01. The horizontal axis represents the culture time of YS01 (**b**) Biodegradation kinetics of CHOL catalyzed by the extracellular crude enzymes of YS01. The horizontal axis represents the catalytic time of the extracellular enzymes. Data in the figure are the average values of three biological repeat experiments and are expressed as means ± SD.

The initial CHOL of 1.00 g/L was biodegraded to 0.26 g/L within 5 d by the cells of YS01, with a removal rate of 74 ± 0.9%, while the loss rate of CHOL in the negative control group within 5 d was only 4.0% (Figure 2a). During the biodegradation, the OD_600_ value reached a maximum of 1.50. Initial CHOL of 0.50 g/L was reduced to 0.13 g/L by the extracellular crude enzymes of YS01 within 24 h, with a removal rate of 74 ± 2.0%, while the loss rate of CHOL in the negative control group within 24 h was only 1.6% (Figure 2b). The specific activity of extracellular crude enzymes was calculated to be 3.49 U/mg. ANOVA was used to validate significance, and there was significant difference between the experimental group and the control group (*p* < 0.05).

### 3.3. Genomic Analysis and Functional Annotation of YS01

To elucidate the biological mechanism underlying CHOL biodegradation by YS01, paired-end sequencing of the draft genome was performed using the Illumina HiSeq platform. Genome annotation predicted a total of 4031 genes with an average GC content of 72.97%. The cumulative length of all genes spanned 4.42 Mbp, including a maximum gene length of 1.02 Mbp and an average gene length of 2.33 Mbp. A total of 3967 protein-coding genes were predicted, accounting for 90.89% of the total genes. Additionally, 60 tRNAs and 3 rRNAs were identified through genome annotation.

Functional annotations from multiple databases were systematically integrated at the gene level to enable multidimensional functional characterization. On the basis of protein sequence alignment, gene sequences were compared with six databases to obtain the corresponding functional annotation information (Table 1).

**Figure 3:**
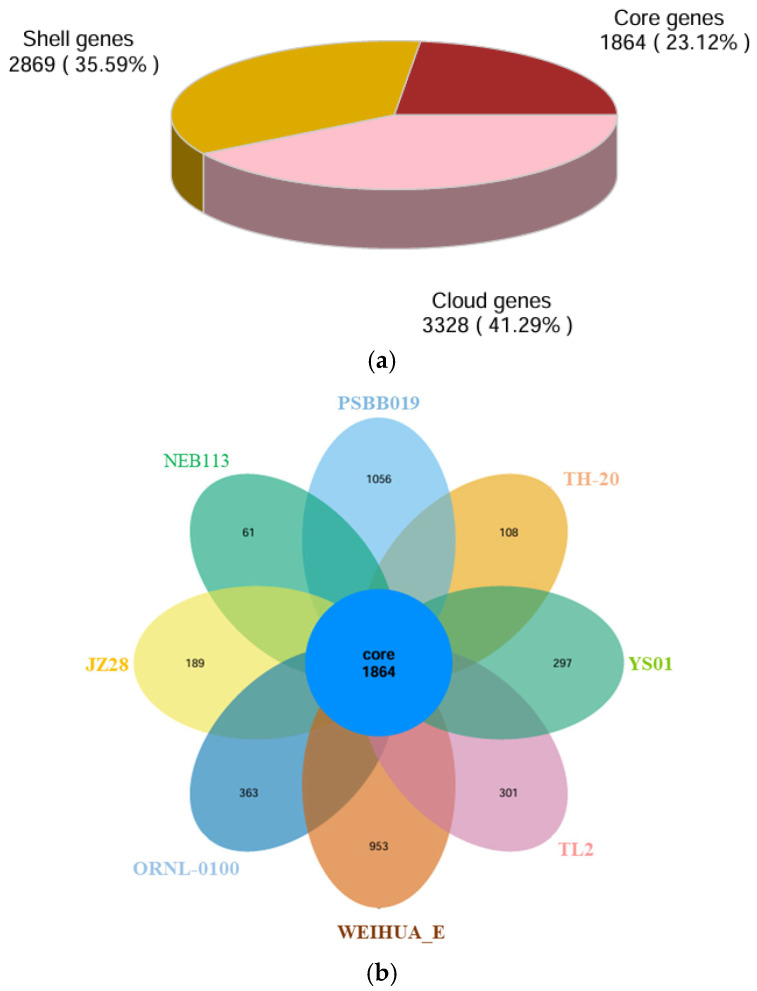
(**a**) Pie chart of homologous gene distribution. (**b**) Comparative genomic analysis of the core genomes of YS01 and other closely related strains.

The genomic data and gene sequences of YS01 were acquired from the NCBI RefSeq (https://www.ncbi.nlm.nih.gov/, 5 March 2025) database taking phylogenetically related strains as reference. The common genes and specific genes of YS01 and related strains were obtained using Roray (https://sanger-pathogens.github.io/Roary/, 5 March 2025) and analyzed in depth. The pie chart of homologous gene distribution visually represents the proportion of core genes, accessory genes, and specific genes. The results show that the number of core genes in YS01 is 1864, accounting for 23.12%; the number of shell genes is 2869, accounting for 35.59%; and the number of cloud genes is 3328, accounting for 41.29% (Figure 3a). The flower plots of homologous genes visually represent the shared and unique genomes among these strains (Figure 3b). The central intersection region shows that the core genome shared these eight related strains, which contains 1864 genes. Furthermore, this figure also identified the unique genes presented in YS01. There are a total of 297 unique genes in YS01 and there are also unique genes in the genomes of other compared strains.

**Figure 4:**
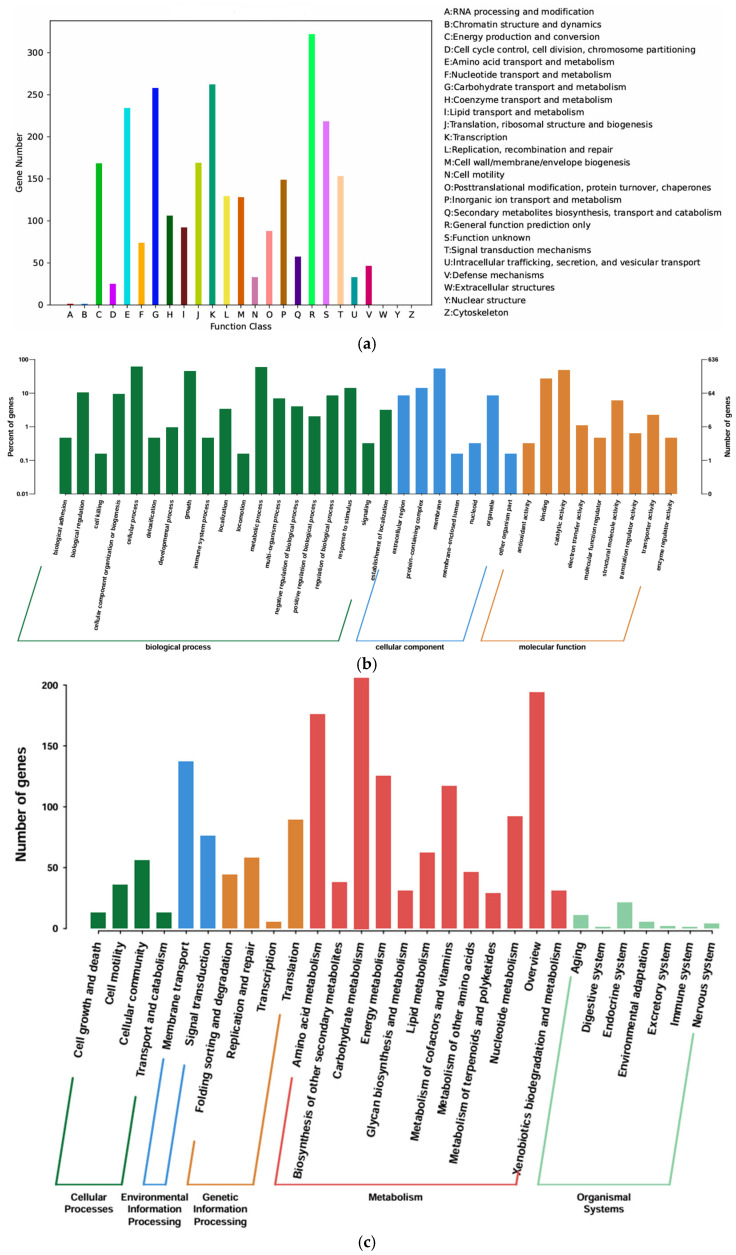
(**a**) COG gene annotation classification of *C. cellulans* YS01. (**b**) GO gene annotation classificatuion of *C. cellulans* YS01. (**c**) KEGG annotation classification of *C. cellulans* YS01.

A total of 2468 genes in the genome of YS01 were annotated in the COG database and classified into 23 functional categories. The annotated genes accounted for 62.21% of the total gene count. Specifically, 234 genes were found to be associated with amino acid transport and metabolism (E), accounting for 9.48% of the total. A total of 258 genes were found to be annotated with respect to carbohydrate transport and metabolism (G), accounting for 10.45% of the total. A total of 324 genes were found to be associated with transcription (K), representing 10.62% of the total. A total of 74 genes were annotated as relating to the transport and metabolism of nucleotides (F), accounting for 3.00% of the total. Concurrently, 169 genes were annotated as relating to translation, ribosome structure, and biogenesis (J), accounting for 6.85% of the total (Figure 4a).

Based on the annotations of the GO database, proteins encoded by the YS01 genome were categorized into three functional classes: molecular function, cellular component, and biological process. A total of 1458 genes were identified as being associated with biological processes, accounting for 57.11% of the total. A total of 543 genes were identified as being associated with cellular components, accounting for 21.27% of the total (Figure 4b). The GO database shows that genes related to catalytic activity, binding, cellular components, cell structures, metabolic processes, and cellular processes are predominantly represented in YS01.

**Figure 5:**
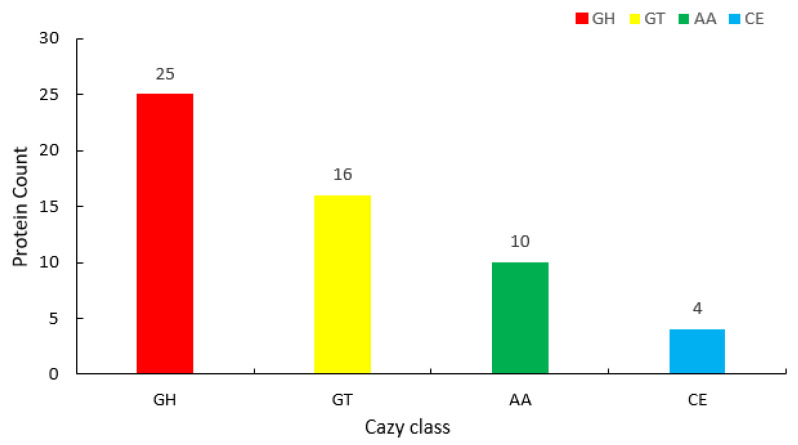
CAZy classification annotation results of YS01.

Additionally, 1043 genes of *C. cellulans* YS01 were annotated in the KEGG database; five colors represent five distinct branches, each of which is further subdivided into different secondary classifications. The vertical coordinate denotes the gene type, while the horizontal coordinate represents the number of genes. Among all KEGG pathways, the majority of genes are involved in amino acid metabolism, membrane transport, and carbohydrate metabolism pathways (Figure 4c). A total of 55 genes encoding carbohydrate active enzymes (CAZy) were identified in the CAZy annotation (Figure 5). The analysis of CAZy revealed that the genome of YS01 contained 25 genes encoding glycoside hydrolases (GHs),16 genes encoding glycoside transferases (GTs), 4 genes encoding carbohydrate esterases (CEs), and 10 genes encoding auxiliary activities (AAs).

**Figure 6:**
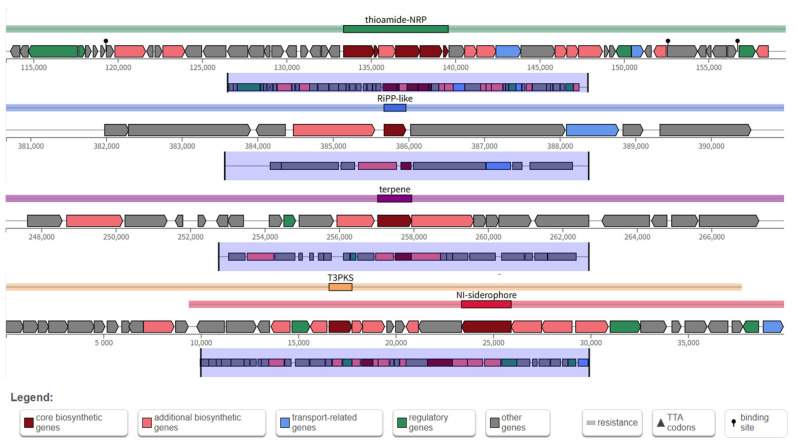
Secondary metabolite biosynthesis gene clusters in YS01.

The antiSMASH software (https://antismash.secondarymetabolites.org/, 10 April 2025) was used to predict secondary metabolite gene clusters in the YS01 genome, identifying four clusters: thioamide-NRP, Type III polyketide synthases (T3PKS), RiPP-like, and terpene biosynthesis pathways. Among the secondary metabolites produced by YS01, alkylresorcinols (ARs) exhibited 100% sequence similarity to a reference sequence, enteromycin exhibited 12% sequence similarity to a reference sequence and terpene exhibited 5% sequence similarity to a reference sequence (Table 2). In YS01, the thioamide–NRP cluster is located between 113,379 and 159,582 nucleotides on the chromosome in region 4.1, spanning a total of 46,204 nucleotides. The Ripp-like cluster is located between 380,676 and 390,966 nucleotides on the chromosome in region 4.2, spanning a total of 10,291 nucleotides. The terpene cluster is located between 247,037 and 267,951 nucleotides on the chromosome in region 5.1, spanning a total of 20,915 nucleotides. The T3PKS cluster is located in region 6.1 of the chromosome and spans a total of 39,930 nucleotides. This gene cluster is made of a series of key genes, including core biosynthesis genes, regulatory function genes, additional biosynthesis genes, and transport-related genes (Figure 6).

### 3.4. Metabolic Pathways and Enzyme of CHOL Biodegradation of YS01

**Figure 7:**
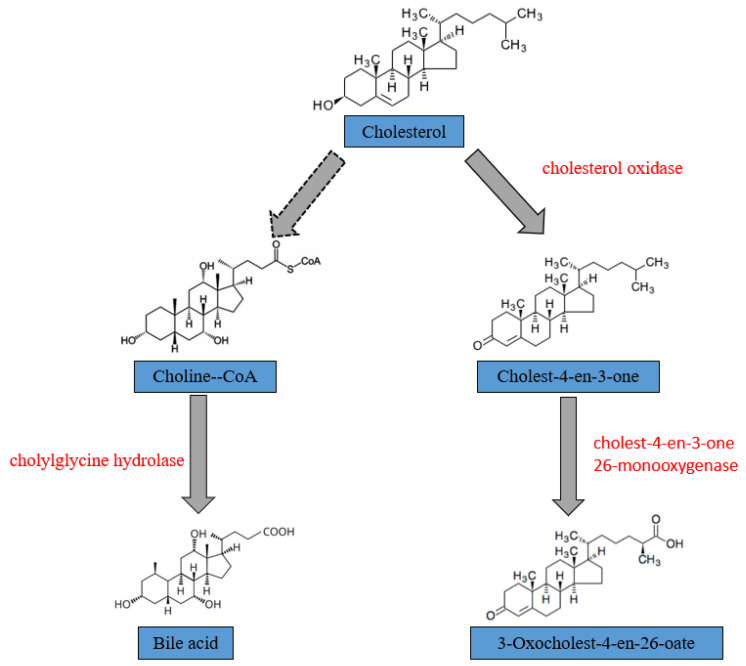
The metabolism pathway for biodegrading CHOL by YS01. The dashed arrow indicates that multiple steps of the reaction are omitted in the middle.

Based on the analysis of GO and KEGG databases, the genes and enzymes involved in the conversion pathway of CHOL were annotated (Table 3). We annotated cholesterol oxidase encoded by the gene *choD* and choloylglycine hydrolase encoded by the gene *cbh* through analyzing the KEGG database. In this study, the biodegradation of CHOL occurs in two pathways. In the passway on the left, CHOL was biodegraded to choline-CoA by a series of enzymes. Finally, under the action of cholinylglycine hydrolase, choline-CoA was catalyzed into bile acid. In another biodegradation pathway, CHOL are converted by cholesterol oxidase to cholest-4-en-3-one, then cholest-4-en-3-one were converted to 3-oxocholest-4-en-26-oate by cholest-4-en-3-one 26-monooxygenase (Figure 7).

## 4. Discussion

*C. cellulans* is a multifunctional strain that can not only hydrolyze gelatin, starch, and protein, but also utilize pectin and cellulose to decompose chitin and has the ability to fix nitrogen [30]. It shows potential application prospects in the treatment of environmental pollution. Mishra et al. [31] reported that *C. cellulans* has special biodegradation capabilities for various organic pollutants carbon or nitrogen sources to maintain growth and metabolic activities. *C. cellulans* is an efficient biodegradation agent and shows great potential in the bioremediation of oil-contaminated sites. Nkem et al. [32] isolated *C. cellulans* DSM 43,879 from oil-polluted tar balls collected in the coastal waters of the Malay Peninsula, which demonstrated the ability to biodegrade 64.40% of biodiesel-related alkane hydrocarbons within a 10 d incubation period. Additionally, *C. cellulans* has been found to have good application prospects in promoting the exploitation of waste resources and the recycling of agricultural waste treatment. Cui [33] effectively leveraged the bioactive properties and substrate biodegradability of *C. cellulans* to mitigate dry matter loss during corn stover ensilage, which consequently minimized biogas byproduct formation and enhanced methane production efficiency by 11.20–21.10%. *C. cellulans* can also be used as plant protectants or fertilizer additives, and have good development value and future development prospects [34]. Ali, Niki, and Iwasaki successfully isolated chitinases from *C. cellulans* SN20 and NTK2, demonstrating the significant potential of these microorganisms in chitinase development [35,36]. It has been found that *C. cellulans* can also be used to produce single-cell protein, bacteriolytic protein, and trehalose [37,38]. In this study, we discovered for the first time that YS01 has the ability to biodegrade CHOL.

In addition, in order to evaluate the CHOL-degrading effect of YS01, we conducted in vitro degradation assays. The results indicated that YS01 indeed has a strong ability in the biodegradation of CHOL. It showed that *Enterococcus faecium* YY01 degraded 50% of CHOL after 5 d of incubation [39]. *Lactiplantibacillus plantarum* SPS109 displayed a CHOL biodegradation rate of 10.89% after 48 h of incubation [40]. *Lactobacillus gasseri* TF08-1 exhibited a strong removal of CHOL (84.40%) within 72 h in vitro [41]. In contrast, Belviso et al. [42] reported that 6 strains of *L. plantarum* and 2 strains of *L. paracasei* failed to biodegrade CHOL. Compared with other reported CHOL-biodegrading strains, YS01 is a promising strain for biodegrading CHOL. Both the cells and the extracellular crude enzymes of YS01 demonstrated efficient CHOL biodegradation activity, indicating their potential for effective CHOL removal. Currently, the reported specific activities of cholesterol oxidase range from 0.5 to 7 U/mg, and the extracellular crude enzymes of YS01 demonstrated certain catalytic activity. We admit that further in vivo experimental studies are still needed, which may reveal more mechanisms and knowledge.

We conducted genomic sequencing to deeply explore functional genes with the aim of analyzing the biological characteristics of YS01. Through the analysis of the COG and KEGG databases, we found that the majority of genes are involved in amino acid transport and metabolism, membrane transport, and carbohydrate transport and metabolism, indicating that YS01 has strong reproductive ability, vigorous biochemical metabolism, and enhanced membrane permeability. This provides a basis for the mass production of functional enzymes. Carbohydrate active enzymes (CAZy) are composed of a series of enzymes with the functions of decomposing, modifying, and biosynthesizing glycosidic bonds, and play an important role in the decomposition of carbohydrates and energy production [41]. We have annotated various carbohydrate-related enzymes in YS01, including GHs, GEs, GTs, and AAs. Furthermore, we predicted that multiple secondary metabolites of YS01, ARs might reduce CHOL levels. Oishi et al. studied the effect of wheat bran ARs on diet-induced metabolic disorders in obese mice and found that ARs could effectively inhibit the absorption of CHOL in the intestine, thereby reducing the concentration of CHOL in the blood [43], which indicates that YS01 has the ability to biodegrade CHOL. Existing studies have shown that ARs has significant potential in cancer prevention, especially in the prevention and treatment of colon cancer [44]. However, the actual function of ARs found in different organisms remains to be further investigated.

To better understand how YS01 works on CHOL, we analyzed the biodegradation pathway of YS01 based on genomic sequencing, which annotated to the two key enzymes. Cholylglycine hydrolase, a member of the BSH family, plays a critical role in CHOL biodegradation by hydrolyzing conjugated bile acids [45]. Bile acid can be metabolized by the microbiota into secondary bile acids in the gut to complete CHOL biodegradation. Cholesterol oxidase is a monomeric bifunctional flavoprotein containing flavin adenine dinucleotide (FAD), primarily produced by Gram-positive and Gram-negative bacteria [46]. The mechanism of biodegrading CHOL may be different among different strains. Compared with some studies, BSH is regarded as the main mechanism for biodegrading CHOL. The ability of three potential probiotics to biodegrade CHOL, *Bacillus flexus* MCC 2458, *Bacillus flexus* MCC 2427, and *Bacillus licheniformis* MCC 2514, was studied by Papanna Shobharani. *B. flexus* MCC 2427 biodegraded CHOL through cholesterol oxidase, while *B. licheniformis* MCC 2514 and *B. flexus* MCC 2458 removed CHOL through assimilation and co-precipitation mechanisms, respectively [47]. Yang et al. studied the CHOL biodegradation pathway of *E. faecium* YY01: Bile acids were produced through the catalysis by N-acyltransferase and choloylglycine hydrolase, thereby achieving the purpose of removing CHOL [39]. In contrast, YS01 possesses the mechanisms of biodegrading CHOL by both BSH activity and cholesterol oxidase activity.

Generally, the catalytic process of cholesterol oxidase mainly consists of two steps. The first step is dehydrogenation: The hydroxyl group at carbon 3 of the CHOL molecule is oxidized to form cholest-5-en-3-one via enzyme-catalyzed oxidation. During this process, the oxidized flavin molecule is reduced by accepting two electrons under the catalysis of enzymes. The reduced flavin immediately reacts with oxygen, generating hydrogen peroxide through a heterocleavage mechanism, while the flavin itself is oxidized. The second step is isomerization: The intermediate cholest-5-en-3-one, formed through oxidation, undergoes enzyme-catalyzed isomerization to form cholest-4-en-3-one. Among these two steps, the dehydrogenation reaction in the first step is the rate-limiting step of this catalytic process [48]. Compared with the dissociation rate of the intermediate cholestol-5-ene-3-one from the enzyme active site, its isomerization reaction rate is faster [49]. In our study, we have annotated the cholesterol oxidase that biodegrades CHOL, which is consistent with the above mechanism.

## 5. Conclusions

*C. cellulans* YS01, a promising bacterial strain with the ability to biodegrade CHOL, was successfully isolated from healthy human intestinal microbiota. We have demonstrated that both the cell and the extracellular crude enzymes of YS01 have a strong removal effect on CHOL. Meanwhile, genomic analysis of YS01 revealed that cholesterol oxidase and choloylglycine hydrolase may play key roles in the conversion of CHOL, which is encoded by gene *choD* and gene *cbh*. The research results of YS01 provide fundamental research value for subsequent in-depth exploration. In the future, we will further conduct safety evaluations and cytotoxicity tests for YS01 to lay the foundation for in vivo experiments.

## Figures and Tables

**Table 1:** The number of functional gene annotations of YS01 from different databases.

Database	Number of Genes
NR	3922
COG	2468
CDD	1606
PFAM	2318
GO	636
KEGG	1043

**Table 2:** Biosynthesis gene clusters of secondary metabolites in YS01.

Region	Type	From	To	Similarity	Most Similar Known Cluster
4.1	thioamide-NRP	113,379	159,582	12%	enteromyic
4.2	Ripp-like	380,676	390,966	—	—
5.1	terpene	247,037	267,951	5%	7-deoxypactamycin
6.1	T3PKS	1	39,930	100%	alkylresorcinols

**Table 3:** Genes and enzymes involved in the biodegradation of CHOL in YS01.

Gene Name	Databes	Enzyme
*choD*	KEGG	cholesterol oxidase [EC:1.1.3.6]
——	GO	cholest-4-en-3-one 26-monooxygenase
*cbh*	KEGG	choloylglycine hydrolase [EC:3.5.1.24]

## Data Availability

The original contributions presented in this study are included in the article. Further inquiries can be directed to the corresponding author.
